# CerebralWeb: a Cytoscape.js plug-in to visualize networks stratified by subcellular localization

**DOI:** 10.1093/database/bav041

**Published:** 2015-05-07

**Authors:** Silvia Frias, Kenneth Bryan, Fiona S. L. Brinkman, David J. Lynn

**Affiliations:** ^1^Department of Molecular Biology and Biochemistry, Simon Fraser University, Burnaby, BC V5A 1S6, Canada, ^2^EMBL Australia Group, South Australian Health and Medical Research Institute, Adelaide, SA 5000, Australia and ^3^School of Medicine, Flinders University, Bedford Park, SA 5042, Australia

## Abstract

CerebralWeb is a light-weight JavaScript plug-in that extends Cytoscape.js to enable fast and interactive visualization of molecular interaction networks stratified based on subcellular localization or other user-supplied annotation. The application is designed to be easily integrated into any website and is configurable to support customized network visualization. CerebralWeb also supports the automatic retrieval of Cerebral-compatible localizations for human, mouse and bovine genes via a web service and enables the automated parsing of Cytoscape compatible XGMML network files. CerebralWeb currently supports embedded network visualization on the InnateDB (www.innatedb.com) and Allergy and Asthma Portal (allergen.innatedb.com) database and analysis resources.

**Database tool URL:** http://www.innatedb.com/CerebralWeb

## Introduction

A key goal of systems biology is to understand the emergent properties of molecular interaction networks and to investigate how these properties are dysregulated in disease. Network visualization tools, such as the popular Cytoscape application (1), allow researchers to interactively visualize these networks and to discern biologically meaningful patterns that may otherwise be difficult to detect. A critical challenge for the visualization of biological networks is to layout the network in a manner more familiar to a biologist. In particular, many biologists think of pathways or networks as having a relatively linear underlying structure with a top (cell surface), middle (cytosol) and bottom (nucleus). Cerebral (‘Cell Region-Based Rendering and Layout’) is an open-source plug-in for Cytoscape that uses subcellular localization information to layout networks in a more biologically intuitive and layered fashion (2). Currently, however, databases that utilize Cerebral, such as InnateDB.com (3, 4), must do so via a Java Web Start version of Cytoscape, which is slow to load, often subject to accessibility issues and generally prohibits the fast and fluid interactivity required by web applications. To address these limitations we have created CerebralWeb, a light-weight, web-embedded, JavaScript implementation of the Cerebral concept, which is intended to support network visualization on systems biology and molecular interaction database websites.

## Approach

CerebralWeb has a number of advantages over the Java Web Start approach of incorporating Cerebral into web applications. First, the embedded nature and speed of CerebralWeb allows it to be seamlessly integrated into interactive websites. This improves usability for desktop browsers but also adds support for increasingly prevalent mobile browsers. CerebralWeb can also interact with other components within the host webpage such as a tabular view of results, a search box or real-time filtering features. CerebralWeb does not require the installation of Java and is not prone to Java related security and accessibility issues that may prevent the execution of Java Web Start dependent applications. The slow execution time of Cerebral visualizations via the Java Web Start also hampers interactivity, especially where a user may wish to perform a rapid series of repeated queries and visualizations, such as when searching a molecular interaction database.

CerebralWeb can be used to visualize any large network (up to ∼2000 nodes for optimal performance given current JavaScript engines and hardware speeds) where the nodes have an attribute value matching one of Cerebral’s subcellular localizations. The user may specify subcellular localization via the ‘localization’ node attribute in an uploaded network file. Alternatively, CerebralWeb may fetch this information in real-time from InnateDB.com (3) via a web service that provides Cerebral subcellular localization annotations for human, mouse and bovine genes. This feature also represents an advance over the Cerebral Desktop application, which requires such annotations to be pre-defined within user supplied network files. To access this feature of CerebralWeb the user need only provide Ensembl, Entrez Gene, UniProt or InnateDB identifiers as node attributes. The web service query will then look-up the gene ontology (GO) (5) Cellular Compartment terms associated with each gene and, using a manually curated map of GO to Cerebral Localization terms, will map the Gene IDs to Cerebral localizations. More information about the web service can be found on the project website and demo pages.

## Implementation

The CerebralWeb plug-in is designed to be easily integrated into any website. The configuration needed to install the plug-in has been reduced to a minimum by providing default settings and extensive code examples that can be reused and adapted if needed. CerebralWeb requires jQuery and is compatible with the current version (v2.3) of Cytoscape.js, an open-source graph library available at http://cytoscape.github.io/cytoscape.js (6). Of the several network visualization packages available in JavaScript (see http://biojs.io/) Cytoscape.js is by far the most mature, widely used and amenable to plug-in development. CerebralWeb is compatible with current versions of major browsers (Chrome, Firefox, Safari and Internet Explorer) and includes a range of ‘out-of-the-box’ interactive features to support even a novice developer in getting embedded visualizations up and running quickly. CerebralWeb rendering speeds are between 4.1 and 7.2 times faster than the Cerebral Desktop Cytoscape plug-in (run via Java Web Start) for small and large networks (see the project website for full benchmarking results). CerebralWeb is also unaffected by execution issues, such as ‘executable’ file blocking and ‘unsigned application’ security popups, which further reduce the usability of the Java Web Start approach in practice.

CerebralWeb is comprised of three JavaScript modules. The first module contains a parser for Cytoscape compatible XGMML files. The parser will also interpret any custom layering information contained within this XGMML graph file or if absent will request subcellular localization annotations via the web service. This module also supports the implementation of an optional ‘dropbox’ feature, that constructs a webpage embedded area into which users may easily ‘drag-and-drop’ XGMML files for automatic parsing and subsequent visualization. The second module gathers the parameters for all the customizable settings in the horizontal layout to generate a Cerebral-like view of the network. Customizable features include the name of each layer, the graph node attribute to which it maps, and its colour and typography, the background colour of the viewer, the colour and width of the grid lines and the colour of inactive and highlighted edges. The third module creates the horizontal layers and places the nodes in each layer based on the node layer attribute. This module can also be implemented to layout nodes according to arbitrary horizontal layers, giving flexibility to developers to generate custom layouts based on other annotations (e.g. biological process, chromosomal location, molecular function, etc.) or to extend the software for use with other types of networks. This module also enables interactive features such as highlighting nodes and edges and displaying names of molecules and interactions. The module also contains a real-time filtering feature that enables CerebralWeb to interact with other components in the webpage. For example, one can configure the network visualization to automatically change as data in a table on the same webpage is filtered. More information to support the implementation of CerebralWeb, which is available under the GNU GPL licence V2, including a tutorial (see ‘Getting Started’) and demo pages, can be found on the project website (http://www.innatedb.com/Cerebral Web).

## Case study

**Figure 1.**
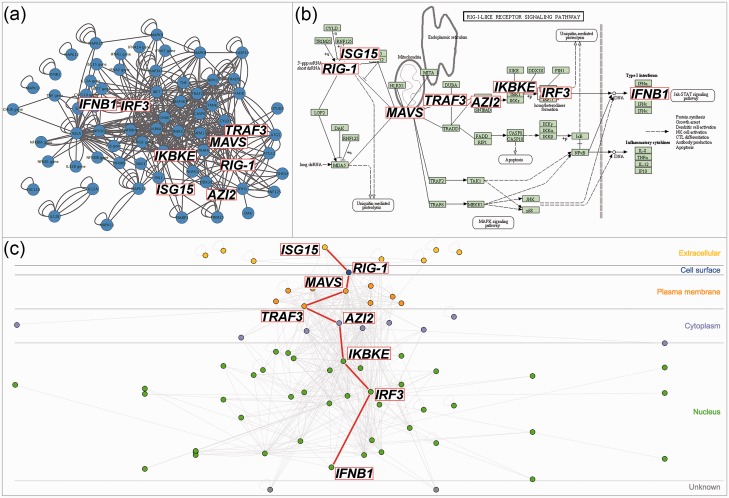
CerebralWeb visualization of the RIG-I pathway. Standard network layouts (**a**) fail to adequately convey the direction of signal flow of the RIG-I pathway as represented by the KEGG pathway layout (**b**). CerebralWeb (**c**) visualizes the molecular interaction network in a more biologically intuitive manner.

InnateDB (www.innatedb.com) and the Allergy and Asthma Portal (allergen.innatedb.com), two databases and analysis platforms for immune-relevant molecular interaction networks, currently use this plug-in to offer visualizations of molecular interaction search results (3, 4). These searches query the internal database to gather the interactions, molecules and their subcellular localization and build the list of nodes and edges on the server side. This list is sent to the client browser and CerebralWeb builds the interactive network visualization. InnateDB also has a function to filter interactions simultaneously in the graph and the table. In this way, interactions can be filtered by participant, interaction type, interactor types, cell type, tissue type or species. It takes only seconds to render the network and the interactivity using the mouse-over feature or when clicking on nodes or edges is close to instantaneous. Figure 1 is the result of a search for the interactions in the ‘RIG-I-like receptor signalling pathway (KEGG)’ in InnateDB. CerebralWeb renders the interaction network in a more biologically intuitive manner than standard network layouts and better captures the direction of signal flow in the pathway.

## Conclusion

CerebralWeb enables the fast and interactive visualization of molecular interaction networks stratified based on subcellular localization or other user-supplied annotation and is designed to be easily integrated into any systems biology or molecular interaction website to support customized network visualization.
